# Prolonged Exercise of the Vocal Organs, and of the Organs of Respiration, as a Means of Strengthening Them, and of Warding off Incipient Phthisis Pulmonalis

**Published:** 1852-10

**Authors:** Stephen W. Williams

**Affiliations:** Deerfield, Mass.


THE NORTH-WESTERN

MEDICAL AND SURGICAL JOURNAL.

NEW SERIES.

VOL. I. OCTOBER, 1852. No. 6.

### ORIGINAL COMMUNICATIONS.

ARTICLE I.—*Prolonged Exercise of the Vocal Organs, and of the Organs of Respiration, as a means of strengthening them, and of warding off Incipient Phthisis Pulmonalis.* By STEPHEN W. WILLIAMS, M. D., Deerfield, Mass.

A DOCTRINE has been very generally inculcated by medical men and others, that in weakness of the breast, lungs and vocal organs, these organs must be kept in a quiescent state, and be but little exercised. This opinion appears to me to be ill founded, and productive of more harm than good. It is contrary to the fact that all the muscles of the body gain strength by exercise;—the arm of the blacksmith obtains power and vigor by constantly using the sledge and the hammer; the general muscular system is invigorated by constant exercise in the open air; and I maintain that the vocal organs are strengthened in the same way. True, *excess* of action may weaken and debilitate them, in the same way that excess of action may weaken the muscular energy of the blacksmith and farmer. It is the extreme which must be avoided.

In illustration of the truth of my position, that exercise of the vocal organs, and of the organs of respiration, strengthens them, and even invigorates the lungs, you will permit me to give some account of my own case, and to refer to the cases of some others.

In the spring of 1824, in consequence of a severe cold, I was most violently seized with pneumonia and bronchitis, which ultimately terminated in chronic inflammation of the bronchia. I was freely bled, which was a great injury to me, as, in a few hours, it induced almost a death-like syncope and palpitation of the heart, from the effects of which I hardly recovered during the succeeding summer, and it did not in the least relieve me of the irritation in my bronchia. The sensation for weeks and months was, most of the time, similar to that of having swallowed something through the trachea. A year or two before I had suffered some from the asthma, and I was now laboring somewhat under that affection, especially upon exercise. I expectorated but little, but my cough was incessant, often keeping me awake all night. I emaciated rapidly, and my friends thought I was sinking into phthisis pulmonalis. In some respects I was better in the fall, but my cough still continued, and the irritation of the bronchia was as severe as ever. I was then a Professor in the Berkshire Medical Institution, and greatly feared I should not be able to give my course of lectures there that fall; I, however, determined to make the attempt, and I went to the College and gave two lectures a-day for many days, and afterwards one lecture a-day, till my course was finished, before a class of more than one hundred students, and in a bad sounding lecture room. I always speak quite loud in lecturing and in reading, and each of my lectures occupies an hour; I was satisfied that my speaking did me no harm, notwithstanding the inflammation of the bronchia still continued. On my return home I spent most of my winter evenings in which I was not engaged in practice in reading loud to my family, sometimes two hours at a time. My cough and emaciation continued, with but little abatement, for more than three years, but I continued my practice of reading loud and lecturing during that time.

In the year 1827 I was attacked with palpitation of the heart, with irregular action of it, which occasioned a kind of syncope anginosa at indefinite intervals. At the commencement of the complaint, the paroxysms came on as often as once in a fortnight, and sometimes as often as once in a week; these paroxysms generally continued from one to three hours, during which time my pulse was continually intermittent; I was always prostrated by these attacks, and was continually faint during the paroxysms. The only relief I could obtain at the time was from lying on my left side and taking stimulants. I always had asthmatic breathing during the period of the attacks, and I think I may date my permanent attacks of the asthma from the time of my bronchitic attack in 1824. I believe I have labored under an organic or structural disease of the heart from the year 1827, which for many years, I am satisfied, has been valvular. Since the year 1839 my pulse has been continually intermittent, at all times, day and night, whether my body was at rest or in motion. No one, who has an organic affection of the heart, is exempt from the asthma. I cannot ascend a flight of stairs, or walk up the smallest hill without inducing it. Previous to the attack of palpitation, in 1827, I was in the habit of smoking three small cigars a day, one after each meal, which is a trifling amount in comparison with what the merest stripling of the present day is in the habit of using. Apprehending that by smoking I might aggravate my complaint, I at once threw aside the use of the cigar, and from that day to this I have not smoked a pipe or a cigar. I am not sure that I received any benefit from this abandonment; but I apprehend, had I continued to smoke, that my complaint would have been aggravated by it. For a few years after this I was in the vile and filthy habit of taking snuff, but I soon became satisfied that it was injuring rather than benefiting me, and I gave it up entirely, and for more than twenty years I have not used a particle of tobacco in any form; nothing would induce me to resort to the pernicious habit again. I more than regained my flesh after giving up the use of tobacco, but the affection of my heart had become so completely structural that it could not be cured by disusing it. Neither do I believe that my complaints were induced by the use of tobacco, yet I have no doubt that it often induces diseases of the heart, and I would recommend a total abstinence from it in affections of that organ. Since I gave it up I am certain that I am not so much affected with fainting fits as before; my general health is better, and for the last twenty years I have done as much business in the practice of physic as I ever did before. Passive exercise, such as riding in a carriage, agrees with me, and I sometimes ride from thirty to fifty miles a-day in the discharge of my professional duties, without inconvenience, over the roughest roads and in the most inclement weather.

I have never yet regained the tone of my lungs, they are always asthmatic, and every time I take cold I suffer greatly from chronic inflammation of the bronchia; still I have constantly kept up the habit of reading loud, and lecturing most of the time, for the last twenty years. In the year 1838 I gave a course of lectures in the College of Physicians of Western New York, and in the same year I gave a course in the Willoughby University of Lake Erie; I gave two lectures a day at each of these places. The next year I gave a course of lectures at the Dartmouth Medical College, and at Willoughby, Ohio. I always spend my leisure winter evenings reading to my family, and I frequently read loud from three to five hours in succession. I am never so happy as when seated in my cushioned elbow-chair, surrounded by my family with their needle-work, by the side of a cheerful fire, and a brilliant solar lamp, reading an interesting book to them; in this way we have all been delighted and instructed, and I am satisfied that my weak lungs have been benefited by the practice.

In addition to the habit of reading loud, I have always been accustomed to expanding my lungs by long inspirations of the breath, and in throwing back my shoulders and arms, to give my lungs the greatest power of expansibility, especially when walking. I never suffer my breast bone to be drawn in, and my lungs to contract, as they otherwise would, from the want of this exertion. To fill the lungs with air to their utmost capacity, needs not the assistance of Ramadge's or Fitche's inhalers, nor, to keep the body erect, of any of those artificial machines to be affixed to the body or chest to obstruct the circulation of the blood; these machines are got up to fill the pockets of the inventors rather than to benefit the invalid.

I was never more forcibly struck with the length of time in which a person could inspire and draw in his breath, and expire and throw it out, than at a meeting of the Teachers' Institute, in Deerfield, last spring. The Professor of Elocution (Russell of Boston) in lecturing upon this subject, exhibited to his class the length of time in which he could be in drawing in his breath and throwing it out; I did not measure the time with my watch, but I absolutely began to grow faint before he got through with the process. Accustomed as I had been to the exercise, I was obliged to desist from following him before he got half through. I afterwards called upon Professor Russell and related to him my experience concerning the use of the vocal organs as a means of strengthening them. He stated to me that he was a Scotchman by birth, and that when he was young he was very puny, and that his lungs were so much affected that his friends hardly expected to rear him; he was, however, early placed at school, and by exercising his vocal organs in reading, by bodily exercise, and the expansion of the lungs, he became, comparatively speaking, a healthy man, and ultimately engaged in the business of a teacher of elocution, where he has occasion to use the vocal organs to a very great extent. He appears to me to have the greatest capacity of voice of any man with whom I am acquainted, although he has a feeble look and is thin in flesh. He appears to be between sixty and seventy years old.

While Professor Russell was relating his case to me, the Rev. Dr. Sears, president of the institute, remarked that there was always in himself a marked predisposition to pulmonary consumption. After he had completed his theological pupilage, and had began to preach, he was attacked with hæmoptysis or bleeding from the lungs, for which he was ordered to journey. He could ride but a few miles in a day. He arrived at a certain place, the name of which has escaped my recollection, on a Saturday afternoon; it was soon known to the villagers that he was there; a committee of the church waited upon him and requested him to preach for them, as the pulpit was vacant; he informed them that he was journeying for his health, and to get rid of preaching, at least for a while. They, however, so urgently insisted upon his taking part in the exercises, that he consented, expecting to be assisted in the prayers, reading the hymns, &c. He preached in the forenoon and found himself no worse, and was urged again to preach in the afternoon and accepted; no inconvenience followed. He pursued his occupation of preaching, and in a few weeks he was ordained as a settled minister over that society, where it is well known his duties as a speaker were of no ordinary character. Few men have more occasion to exercise their vocal organs than Dr. Sears; his lungs appear to be sound; I should think he was near sixty years old.

Our American Hippocrates, the immortal Rush, speaking of preventives of consumption, recommends "the moderate use of the lungs in reading, public speaking, laughing and singing. The lungs when debilitated derive equal benefit with the limbs of the body from moderate exercise." In his paper on the use of common salt in the cure of hæmoptysis, he says, "Those persons who have been early instructed in vocal music and who use their vocal organs moderately through life, are seldom afflicted by an hæmorrhage from the lungs. Lawyers, players, public criers, and city watchmen, all of whom exercise their lungs either by *long or loud speaking*, are less affected by the disorder than persons of other occupations."

In the second volume of the *Journal of Health*, I find the following judicious observations on the voice, corroborating my opinion:—"The voice should never be exercised beyond its strength, nor strained to its utmost pitch without intermission; such mismanagement would endanger its power altogether, and render it hoarse and grating. Frequent changes of pitch is the best preservative. The voice, as well as the health of the speaker, suffers materially unless the chest is allowed to expand freely; hence, all compression or restraint should be carefully removed from this portion of the body; for the same reason an erect position should be assumed, as well in speaking and reading aloud as in singing. The tone of the voice is also considerably impaired, and its strength diminished, by a tightly drawn or large cravat. Both in speaking and singing, therefore, the neck should be free from compression, and but slightly covered. The great means of improving the voice, as of all other improvements, is constant and daily practice. The professional exercises at the bar, in the senate, or in the pulpit, if properly attended to with a view to improvement, may suffice for the orator of our times; but the ancients, besides this, were in the daily practice of preparatory declamation; their rule was, after proper bodily exercise, to begin at the lowest tones of the voice and proceed gradually to the highest. They are said to have pronounced about five hundred lines in this manner, which were committed to memory in order that the exertions of the voice might be less embarrassed. The second rule has been anticipated, which is regular bodily exercise. The ancients recommended walking a certain distance before breakfast—about a mile. Riding on horseback we do not find recommended, or practised as a mere exercise,"

though Dr. Sydenham and Dr. Rush considered it as certain a cure for consumption as bark for intermittent fever.

Enough has now been said upon the subject of the exercise of the vocal organs, as a method of strengthening them. I would, however, before closing my remarks, as I am upon the subject of warding off phthisis, warn the consumptive invalid, *after the disease has fairly fixed itself*, against a change of climate—from a northern to a tropical one. While a *very few*, perhaps, have been benefited by it, thousands have found an early grave there. The debilitating effects of the climate, and the discomforts of absence from home and the endearing society of friends, have too often hurried many a victim to an untimely grave. A resort, too, to the thousand-and-one nostrums for the cure of this insidious complaint has also added myriads to the tomb. A specific remains to be found for the cure of this direful complaint, notwithstanding the vaunted eulogies of the various preparations of iodine, cod-liver oil, phosphate of lime, naphtha, &c., &c.

But to the predisposed, before the complaint has actually invaded, a resort to vocal and bodily exercise, a short summer residence at Mackinac, at Lake Superior, at the prairies of Southern Wisconsin and Northern Illinois, and especially at some of your beautiful lake-ports like Chicago, Milwaukee, &c., now that facilities for visiting those places are so great, cannot be otherwise than salubrious, as affording constant amusement for the mind and sufficient healthy exercise for the body.

DEERFIELD, MASS., *Sept.* 13, 1852.

